# Editorial: Achilles heel of CAR T-cell therapy

**DOI:** 10.3389/fimmu.2025.1724641

**Published:** 2025-10-31

**Authors:** Maurizio Chiriva-Internati, Fabio Grizzi

**Affiliations:** ^1^ Division of Internal Medicine, Department of Gastroenterology, Hepatology and Nutrition, The University of Texas MD Anderson Cancer Center, Houston, TX, United States; ^2^ Department of Immunology and Inflammation, IRCCS Humanitas Research Hospital, Milan, Italy; ^3^ Department of Biomedical Sciences, Humanitas University, Milan, Italy; ^4^ Histology Core, IRCCS Humanitas Research Hospital, Milan, Italy

**Keywords:** CAR-T cell therapy, chimeric antigen receptor, CAR-NK, clinical application, immunotherapy

Chimeric antigen receptor (CAR-T) cell therapy has emerged as a revolutionary treatment modality in clinical oncology and particularly in the management of haematological malignancies such as B cell acute lymphoblastic leukaemia, large B cell lymphoma and multiple myeloma, with unprecedented response rates and even some cures in patients with relapsed and/or refractory disease (1). The concept of a chimeric T cell receptor, which combines antibody-derived variable regions (VH/VL) with T cell receptor (TCR)-derived constant regions, was first reported in 1987 by Japanese immunologist, Dr. Yoshikazu Kurosawa, and his team at the Institute for Comprehensive Medical Science in Aichi, Japan (2). In 1989, Dr. Zelig Eshhar and his team at the Weizmann Institute developed a chimeric T-cell receptor (cTCR) that enabled T cells to recognize antigens in an MHC-independent manner by fusing antibody-derived variable regions with T-cell receptor constant regions. Although the cTCRs were functional and capable of activating T cells, their double-chain structure required dual viral transduction, resulting in low efficiency. To overcome this, Eshhar’s group engineered a single-chain chimeric receptor, later termed the first-generation CAR, that combined an antibody’s single-chain variable fragment (scFv) with intracellular signaling domains, simplifying expression and enhancing antigen-specific, MHC-independent T-cell activation. The first-generation CARs consisted of a single-chain variable fragment (scFv) fused to CD3ζ or FcϵRIγ, and these engineered T cells demonstrated anti-cancer activity in murine models. Encouraged by promising preclinical findings, the first human CAR T-cell trials targeted ovarian and metastatic renal cell carcinomas using autologous T cells engineered to express MOv-γ and scFv(G250) chimeric receptors, respectively. Although these treatments were well tolerated, they did not reduce tumor burden, likely due to the limited *in vivo* persistence of the infused CAR-T cells. T-cell activation requires two signals: antigen recognition through the TCR–peptide–MHC (pMHC) interaction and co-stimulation via receptors such as CD28. To address this limitation, Dr. Michel Sadelain’s group at MSKCC developed a chimeric receptor incorporating both CD3ζ and CD28 signaling domains, thereby providing dual activation signals that enhanced antigen-dependent proliferation, IL-2 secretion, and cytotoxic activity *in vitro* (3). Second-generation CD19 CAR-T cell therapy has since revolutionized the treatment of B-cell malignancies. However, in chronic lymphocytic leukemia (CLL), response rates and durability remain inferior to those observed in other indolent B-cell lymphomas. In 2024, Derigs et al. reported early clinical results from the HD-CAR-1 trial, evaluating academically manufactured third-generation CAR-Ts in patients with relapsed/refractory CLL (4). Third-generation anti-GD2 CAR-T cells (GD2-CART01) showed promising efficacy in children with high-risk metastatic, relapsed, or refractory neuroblastomas in a phase 1/2 trial. The Locatelli et al. (5) final report results from 54 children, confirming that GD2-CART01 can induce durable remissions in this population.

This Research Topic aimed to tackle the key challenges in developing sustainable and cost-effective CAR-T cell therapies. Its main objectives were to identify promising strategies for reducing manufacturing costs, automate production processes to minimize human error, and enhance the therapeutic efficacy of CAR-T cells. The research also sought to explore how to lower CAR immunogenicity, improve infiltration into the tumor microenvironment, and prevent antigen escape. By addressing these critical areas, the work aimed to advance the field toward more effective, accessible, and safer cancer treatments.

In their manuscript, Harer et al. discussed upcoming strategies and current challenges in designing CARs for recognition of antigen low cancer cells, aiming at augmenting sensitivity and finally therapeutic efficacy while reducing the risk of tumor relapse. Specifically, CAR-T cells are engineered “living drugs” designed to recognize specific tumor antigens and eliminate malignant cells through targeted immune activation. Despite their success in treating B cell malignancies, many patients experience relapse due to antigen loss or T cell exhaustion, which limits long-term efficacy. To overcome these challenges, current research focuses on developing next-generation CARs with enhanced antigen sensitivity, enabling the detection and elimination of cancer cells expressing low levels of target antigens. Cancer cells can evade CAR-T cell therapy by reducing the expression of target antigens, rendering them invisible to immune attack and leading to tumor relapse. Although enhancing CAR-T cell sensitivity to low antigen levels could address this issue, it also raises significant safety concerns, as many tumor-associated antigens are shared with healthy tissues, increasing the risk of on-target off-tumor toxicity. Consequently, ongoing research focuses on balancing efficacy and safety through strategies such as logic-gated CAR designs, cooperative targeting approaches, and the careful selection of tumor-selective antigens to minimize adverse effects while maximizing therapeutic benefits. CAR-T cell therapy has made remarkable strides in treating hematological malignancies. However, the widespread adoption of CAR-T cell therapy is hindered by several challenges. Li et al. comprehensively examined the clinical challenges of CAR-T cell therapy and outlined strategies to overcome them, aiming to chart pathways beyond its current Achilles’ heel. CAR T cell therapies have achieved remarkable success in treating hematologic cancers, yet their broader use remains limited by high costs, long manufacturing times, safety issues, and variable efficacy. Advancements in gene editing technologies and delivery systems are essential to overcome these barriers and redefine the development of next-generation CAR-T cells. As these tools continue to evolve, they hold the promise of creating safer, more potent, and more accessible CAR-T cell therapies, transforming them into routine and affordable treatment options for a wider range of patients. Although CAR-T cell therapy remains one of the most innovative immunotherapeutic approaches with remarkable clinical success, its broader application is limited by lengthy manufacturing times, high costs, and patient-to-patient variability (6). Despite notable advances in the development of universal CAR T (U-CAR T) cells, a stable and standardized cell bank has yet to be achieved. Mohammad et al. systematically reviewed and evaluated the efficacy of modular (universal) CAR T-cell platforms in xenograft mouse models. Across 33 studies encompassing 15 distinct platform designs, modular CAR-T cells were shown to significantly reduce tumor burden and improve survival compared to negative controls, achieving outcomes comparable to conventional CAR-T cell therapies. Overall, these findings suggest that modular CAR T-cell platforms are effective and represent a promising, flexible, and controllable approach for next-generation cancer immunotherapy. Select patients with relapsed/refractory aggressive B cell lymphoma may benefit from bridging radiation (bRT) prior to anti-CD19-directed CAR-T. Manzar et al. evaluated 51 adults with relapsed or refractory diffuse large B-cell lymphoma (DLBCL) who received bridging radiation therapy (bRT) prior to anti-CD19 CAR-T cell therapy. Just over half (51%) received comprehensive bRT to all disease sites, and 29% also received systemic therapy. The overall response rate at 30 days post–CAR-T was 82.4%. Median overall survival (OS) was 22.1 months, and median progression-free survival (PFS) was 7.4 months. One-year OS and PFS rates were 80% and 78%, respectively, while two-year rates were 59% and 54%. Comprehensive bRT was associated with improved OS and PFS (p ≤ 0.04). Poor outcomes were linked to ECOG performance status ≥2, advanced stage (III/IV), high IPI score (≥3), non-GCB histology, and radiation doses ≤30 Gy. Relapse occurred in 51% of patients, with 46% of relapses within the radiation field, especially among those with bulky disease or poor early CAR-T response. Overall, the study concludes that bRT before CAR-T is an effective and feasible strategy for selected patients with aggressive B-cell lymphomas, and that comprehensive radiation to all disease sites improves survival outcomes. CAR-T cell therapy has transformed cancer treatment, but key challenges remain, including antigen loss and optimizing CAR design as discussed by (Gomez-Melero et al.). To address these issues, multi-targeted approaches, such as tandem CAR-T (TanCAR-T) cells, have been developed. These engineered cells can recognize multiple tumor antigens simultaneously, reducing relapse risk and improving treatment efficacy. Preclinical and clinical studies in both hematologic cancers and solid tumors have shown that TanCAR-T therapies are effective, safe, and associated with relatively low relapse rates. Despite these promising results, several challenges persist, such as determining the optimal CAR construct, selecting the best antigen targets, and improving transduction efficiency. Overall, tandem CAR-T cells represent a promising advancement in immunotherapy, with ongoing research needed to refine their design and maximize their clinical benefits.

Li et al. outlined the limitations of CD28-based CAR T-cell therapies, evaluated current strategies designed to optimize CD28-based CAR constructs, and discussed future directions and clinical prospects for enhancing their therapeutic potential. CAR-T cell therapy, which engineers T cells to specifically target cancer cells, has achieved major advances in recent years. Current approved CAR-T products are second-generation designs that include co-stimulatory domains, essential for T-cell activation and function. Among these, CD28-based co-stimulatory molecules provide strong cytotoxic effects but are limited by high relapse rates, short-lived efficacy, and severe side effects. Recent research has focused on improving CD28 function by mutating its signaling motifs, combining co-stimulatory domains, and optimizing other CAR components to enhance anti-tumor activity and minimize toxicity. Yin et al. have explored the use of sodium citrate to reduce exhaustion and enhance the function of CAR-T cells. While CAR-T therapy is effective against blood cancers, its success in solid tumors is limited by T cell exhaustion, often driven by tonic signaling and calcium activity during cell expansion. They generated anti-CD70 and anti-mesothelin (MSLN) CAR-T cells and cultured them with sodium citrate. Results showed that citrate-treated CAR-T cells had reduced exhaustion, higher memory T cell levels, and improved anti-tumor efficacy, both *in vitro* and *in vivo*. Treated CAR-T cells also demonstrated better persistence and lower tumor recurrence. This study highlights the potential of sodium citrate to overcome a major limitation of CAR-T cell therapy in solid tumors. Sodium citrate-pretreated CAR-T cells (CITR CAR-T) showed stronger persistence, greater anti-tumor efficacy, and prevention of tumor recurrence *in vivo* compared to untreated cells. Mechanistic studies revealed that sodium citrate suppresses CamkII phosphorylation, thereby inhibiting mTORC1 signaling and glycolysis, pathways linked to T cell exhaustion. These effects collectively promote the formation of memory T cells and sustain CAR-T activity. Although the experiments were performed in a cell-derived xenograft (CDX) model, which may not fully mimic human tumors, these findings suggest that sodium citrate could be a simple, safe, and cost-effective strategy to improve CAR-T therapy for solid tumors. The study concludes that sodium citrate enhances CAR-T persistence and function by modulating calcium, mTOR, and metabolic signaling, offering a promising avenue for future clinical applications.

De Angelis et al. presented results from a European survey conducted by the T2Evolve Consortium under the EU’s Innovative Medicines Initiative (IMI), which examined current analytical methods employed in CAR T-cell therapy across Europe. Between February and June 2022, a total of 53 respondents from 13 countries, including researchers, manufacturers, and clinicians, completed a 36-item questionnaire addressing quality control of apheresis materials, CAR T-cell drug products, and post-infusion immune monitoring. The results revealed considerable variability in analytical practices among institutions. While most respondents used standard assays for safety and efficacy testing per Pharmacopeia requirements, only a minority performed detailed phenotypic analysis of T-cell subsets or assessed activation and exhaustion markers in final products. This study highlights an urgent need to standardize CAR-T cell functional potency assays and to identify predictive biomarkers for treatment response, relapse, and toxicity. It also found inconsistent CAR-T cell monitoring during patient follow-up. This first pan-European survey provides a snapshot of current CAR-T cell analytical practices and emphasizes the importance of harmonization across centers to improve the safety, efficacy, and accessibility of CAR-T cell therapies in Europe. The survey revealed widespread heterogeneity and lack of standardization across all phases of CAR-T cell therapy in Europe, from apheresis collection to patient monitoring. T2Evolve highlights the need to harmonize analytical methods, quality control assays, lymphodepletion protocols, and immune-monitoring standards to ensure consistent product quality, patient safety, and equitable access to CAR-T cell therapies across Europe. Currently, no standardized protocols exist for leukapheresis collection, quality assessment, or cryopreservation, and only a few centers routinely characterize leukapheresis products. About 66% of respondents emphasized standardization of apheresis and cryopreservation, while identifying biomarkers predicting manufacturing success as a research priority. All centers use viral transduction, but over half (52%) called for standardized assays, including vector copy number, sterility, and flow cytometry tests. Rapid quality testing was also seen as essential to shorten manufacturing time. Most centers use fludarabine and cyclophosphamide (Flu/Cy), though alternatives like Bendamustine are being explored. Lymphodepletion regimens and monitoring practices vary widely, with fewer than half performing additional tests during toxicities, underscoring the lack of consensus on predictive biomarkers such as IL-6, IFN-γ, and IL-1.

Bolsée et al. have investigated a dual-targeting CAR-T cell strategy to address antigen escape, a major cause of relapse in B-cell malignancies such as acute lymphoblastic leukemia (B-ALL). The authors designed tandem CAR-T cells that simultaneously recognize CD19 and NKG2D ligands (NKG2DL), stress-induced molecules commonly expressed on cancer cells but not on healthy tissues. Three tandem CAR constructs were developed, and two demonstrated strong anti-tumor activity against both CD19^+^ and CD19^-^ cancer cells. Compared to conventional CD19 CAR-T cells, these tandem CARs maintained similar cytokine production, cytotoxicity, and proliferation when engaging CD19^+^ targets, while retaining effectiveness against CD19^-^ cells. In experiments with primary B-ALL samples and xenograft models mimicking CD19^–^ relapse, the selected CD19/NKG2DL tandem CAR-T cells successfully controlled tumor growth and prevented relapse. This study provides proof-of-concept that NKG2D-based tandem CAR T-cells can overcome CD19 antigen loss and enhance long-term therapeutic efficacy in B-cell malignancies. The Authors conclude that CD19/NKG2DL tandem CAR-T cells provide a promising strategy to prevent antigen escape, extend therapeutic reach, and maintain anti-tumor efficacy in B-cell malignancies. Furthermore, the broad expression of NKG2DL suggests potential applicability in solid tumors and tumor-associated microenvironments, offering a versatile platform for multispecific CAR T-cell therapies. Muthuvel et al. describes a safer and efficient method for generating anti-CD19 CAR-T cells using self-inactivating (SIN) lentiviral vectors for adoptive immunotherapy. The CAR construct included a CD8α hinge, CD28 transmembrane and co-stimulatory domains, and CD3ζ signaling, and T-cells were pre-activated via CD3/CD28 beads before transduction. The resulting CAR-T cells achieved a transduction efficiency of approximately 28% at an MOI of 10 using high-titer lentiviral vectors (~9.85×10^7^ TU/ml). The T-cells expanded about 148-fold over 12 days in serum-free media, maintaining high viability (>87%) and exhibiting a predominantly CD8+ effector memory phenotype by days 7–12. Functionally, the CAR-T cells demonstrated specific antitumor activity, lysing CD19^+^ NALM6 cells (~28% at a 1:1 ratio) and producing robust antigen-specific responses, including IFN-γ secretion and CD107a degranulation. Safety modifications included removal of WPRE, GFP, and P2A sequences from the CAR construct. Overall, this study establishes a reproducible workflow for generating functional, safe, and scalable anti-CD19 CAR-T cells, suitable for applications in cancer and autoimmune diseases involving CD19^+^ B-lineage cells. 

**Figure 1:**
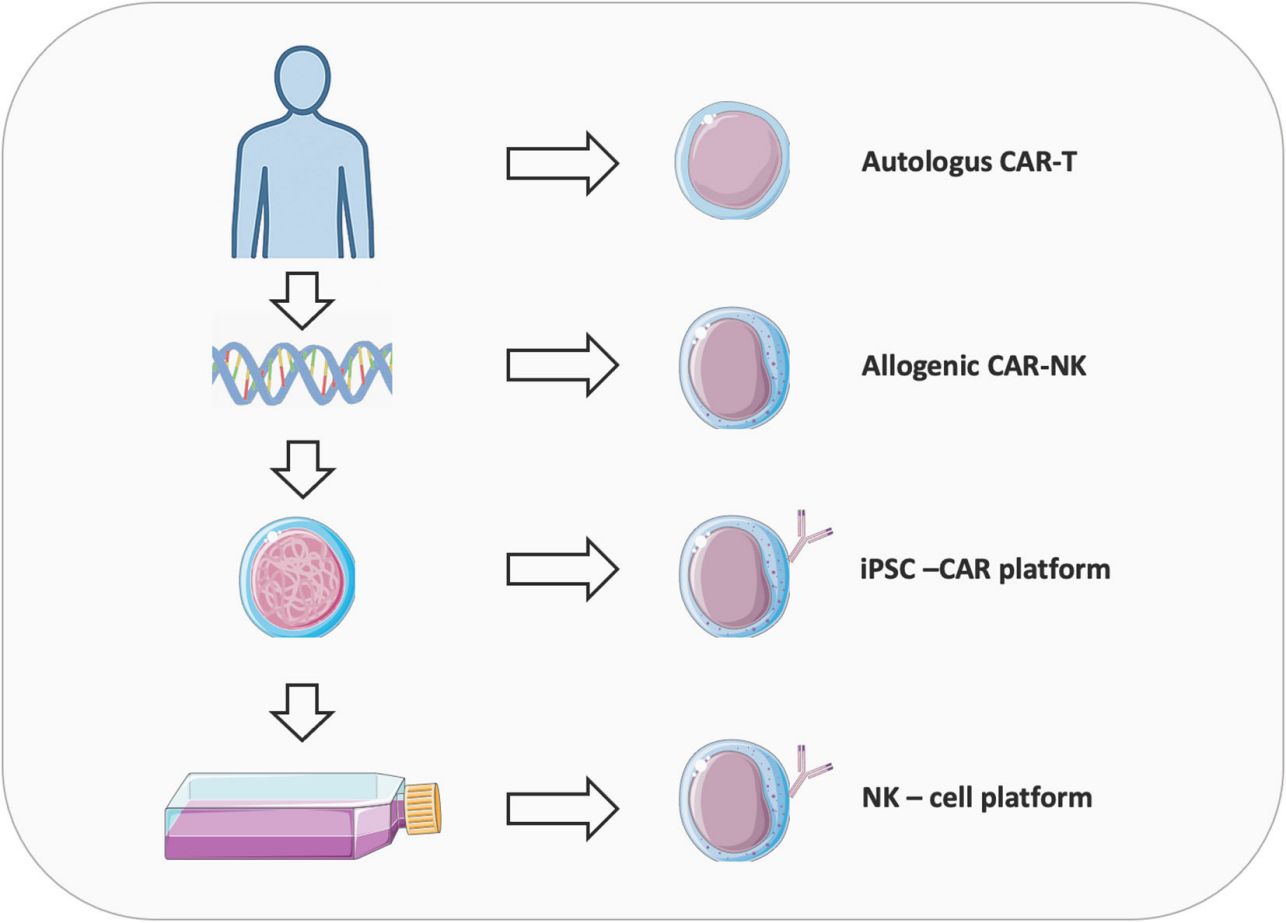
Overview of the evolution of CAR therapies from autologous CAR-T to allogeneic CAR-NK and iPSC-derived platforms. Autologous CAR-T uses the patient’s own T-cells, reducing rejection and “Graft-versus-host” disease risk but face high cost, variable quality, and manufacturing delays. Allogeneic CARs from donors or cell lines offer scalable, standardized production but must overcome alloreactivity and immune rejection, often via CRISPR-mediated TCR/HLA editing. NK cells provide an alternative with innate cytotoxicity, lower CRS risk, and compatibility for allogeneic use, enhanced by NK-specific signaling domains. Induced pluripotent stem cells (iPSCs) enable clonal expansion, gene editing, and differentiation into NK cells with consistent phenotypes, though challenges like differentiation heterogeneity and transgene silencing remain, addressable via safe-harbor knock-ins and transcriptional programming.

CAR-based cell therapies have revolutionized cancer treatment by enabling precise, antigen-specific immune activation against malignant cells (**Figure 1**). Since the first FDA approvals of CD19-directed CAR-T cell therapies in 2017, adoptive cell therapy has progressed from a conceptual innovation to a transformative clinical modality. CAR-T cell therapy utilizes patient-derived T cells genetically engineered to express synthetic receptors that redirect specificity toward tumor-associated antigens (7, 8). This approach has shown curative potential in hematologic malignancies; however, its efficacy in solid tumors remains limited by antigen heterogeneity, immunosuppressive tumor microenvironments, and manufacturing complexity. Globally, over 1,000 clinical trials are investigating strategies to broaden CAR therapy’s reach, including the development of “off-the-shelf” universal platforms. Recent advances in CAR–natural killer (CAR-NK) cells and induced pluripotent stem cell (iPSC)-derived CAR immune cells offer promising avenues to overcome current challenges in scalability, safety, and cost (9–13). CAR-NK cells enable allogeneic use with lower toxicity risk, while iPSC-derived immune cells facilitate the production of homogeneous, programmable effector populations at an industrial scale. Together, these innovations signal a paradigm shift toward universal, programmable, and ethically scalable CAR-based immunotherapies. Supported by CRISPR-mediated gene editing and refined antigen-targeting strategies, next-generation CAR platforms are poised to expand the therapeutic frontier to both hematologic and solid malignancies.
